# Tell the Device Password: Smart Device Wi-Fi Connection Based on Audio Waves

**DOI:** 10.3390/s19030618

**Published:** 2019-02-01

**Authors:** Liang Liu, Zhaoyang Han, Liming Fang, Zuchao Ma

**Affiliations:** College of Computer Science and Technology, Nanjing University of Aeronautics and Astronautics, Nanjing 210016, China; sunrisehan@nuaa.edu.cn (Z.H.); fangliming@nuaa.edu.cn (L.F.); macher@nuaa.edu.cn (Z.M.)

**Keywords:** Internet of things, smart devices, Wi-Fi provisioning, audio waves

## Abstract

IoT devices are now enriching people’s life. However, the security of IoT devices seldom attracts manufacturers’ attention. There are already some solutions to the problem of connecting a smart device to a user’s wireless network based on the 802.11 transmission such as Smart Config from TI. However, it is insecure in many situations, and it does not have a satisfactory transmission speed, which does not mean that it has a low bit rate. It usually takes a long time for the device to recognize the data it receives and decode them. In this paper, we propose a new Wi-Fi connection method based on audio waves. This method is based on MFSK (Multiple frequency-shift keying) and works well in short distance, which enables the correctness and efficiency. In addition, audio waves can hardly be eavesdropped, which provides higher security than other methods. We also put forward an encryption solution by using jamming signal, which can greatly improve the security of the transmission.

## 1. Introduction

Smart devices are getting more and more popular in our daily life. Although they have brought much convenience, they still have many security problems [1]. Most smart home devices are based on wireless networks, and their widely used wireless network may have many security problems. Typically, there is a threat even before these smart devices are used. Some researchers have indicated that the unsafe transmission can lead to password disclosure and other issues [2].

Smart devices usually need to connect to the Internet the very first they are used before they can serve people [3]. Therefore, users should connect the wireless devices to their known Wi-Fi before they can use them. There are three parties in the configuration progress: user, smart device and cloud server. User sends the ssid and password of a wireless network to device with a certain app provided by manufacturers. The device receives the credential and try to connect to the particular wireless network, and then the device can communicate with the cloud server if it connects to the network successfully. When a user is far away from the device, the user sends an order to cloud server and the cloud server forwards it to the device.

Consider the situation that a device has UI (User Interface), under which users can just input the password to it. However, many devices are not with UIs, such as cameras. Then, the problem arises of how to communicate the wireless network information (ssid and password) to a device without UI securely. This problem is similar to securely pairing wearables, to which researchers have presented several solutions (e.g., [4,5]). SmartCfg (SmartConfig) [6] is one of the most popular solutions to deal with Wi-Fi configuration. It works by broadcasting 802.11 packets, and, due to the permission problem, credentials are stored in meta data such as length, destination address, etc. There are three ways to store credentials in SmartCfg: Data in Multicast Addresses (DMA), Data in Packet Length (DPL) and Hybrid. These methods all store data in meta-data. However, Li et al., [7] indicated that storing data in meta-data of Wi-Fi traffic will face great potential threat. Thus, it is dangerous to store data in meta-data of Wi-Fi packets. Although SmartCfg can work properly in most situations, some researchers have already found several ways to attack it and obtained considerable results because of the limitation of SmartCfg on packet encoding [2]. Attackers can easily figure out which method (one of DMA, DPL and Hybrid) the producer is using by analyzing on what SD the device is based [8], which exposes the position of the credential in the packet, and then attackers recover the encoded credential using some techniques such as differential traffic analysis. This problem can be solved if encryption methods are involved. Ghose et al., [9] found executing key agreement protocols is vulnerable to Man-in-the-Middle (MitM) attacks. Wu et al., [10] pointed out existing device pairing schemes vulnerable to MitM attacks. In addition, SmartCfg can hardly be very efficient because of its Wi-Fi transmission. Moreover, 802.11 packets are very easy to be eavesdropped [11], and there are many studies on preventing eavesdropping in IoT recently [12,13,14].

In this paper, we propose a new solution to transmit information that uses audio waves instead of 802.11 packets. Compared to traditional methods, using audio waves can avoid many disadvantages. Unlike 802.11 packets, audio wave can store message in any part of a packet. The format of packet can be designed to fit the secure transmission. Audio waves are also more easy to receive and decode than Wi-Fi packets. Moreover, audio waves have a better secure distance, which means that attackers need to eavesdrop the audio waves from a much closer place than Wi-Fi packets. To use audio waves, microphones are necessary. Nowadays, most smart devices have audio system to receive orders from users. Thus, this requirement is not a problem. Besides, deploying a microphone to a smart home device is quite simple and low cost.

The proposed configuration method’s advantages can be summarized as below:

- Audio wave transmission is not based on 802.11 or some other standards. Service providers can freely design the format their audio packets. It means that credentials are not necessarily stored in meta-data area. Independent packet design also means that we reduce space waste to some extent, and the format of packet can be not public to all, which means encryption can be more secret.
- It is much more difficult to eavesdrop audio waves than Wi-Fi packets. Loudness and noise will interfere with attackers efficiently when they try to conduct attack actions in a secure distance, which is usually not near the device.
- Audio wave transmission has higher efficiency than SmartCfg. It is fast to generate the encoded audio waves and broadcast them. Receiving and decoding also take very little time. Therefore, network connection can be completed very quickly.
- Most devices are already equipped with audio components, which means deploying this new configuration solution would be very simple.

In addition to Wi-Fi configuration, audio wave transmission can also be used in many other fields, such as short distance control. For example, smart locks have appeared in our life for a long time. Users control the door lock using their mobile phone, and the control process can use the audio framework instead of Wi-Fi, bluetooth, ZigBee or other methods [15]. We know that there are many places having weak signal where people cannot connect to wireless network or can only have an unbearably low network speed. With this situation, the audio based message transmission can work well.

This paper is organized as follows. In Section 2, we introduce the background and related works. In Section 3, we present our new audio wave based Wi-Fi credential transmission method and its design. In Section 4, we give the experiments’ results and compare our solution with SmartCfg. Finally, we give our conclusion and future work in Section 5 and Section 6.

## 2. Background and Related Work

### 2.1. Wi-Fi Configuration of Smart Devices

Smart home technology has matured with the development of Internet of Things (IoT) technology. A smart device usually adds some components to a common device to implement some functions. For example, homeowners could control their speakers just by speaking particular words. This feature becomes increasingly practical and functional with the development of speech recognition technology. Homeowners could also send orders such as “turn on”, “turn off” to smart devices by an app remotely. Smart home technology has made people’s daily life more convenient. Thus, research on smart devices nowadays is important.

Most features of smart devices are based on networks, which means that the big premise is having all devices connected to networks. As mentioned above, many devices do not have UIs, as they are usually unnecessary. For example, a door does not need a UI to open or lock. Installing UI only for Wi-Fi connection is quite uneconomical. Therefore, the technology for connecting devices without inputting to wireless networks is valuable.

**Figure 1:**
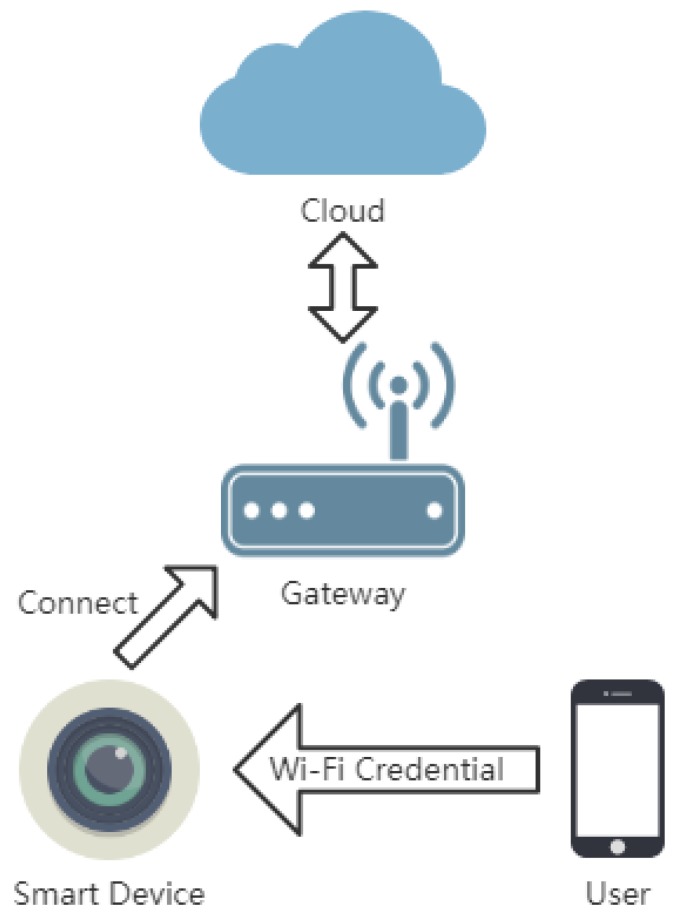
Device–gateway–cloud scheme in smart home. Users need to tell their devices the Wi-Fi credentials so that the smart devices can communicate with cloud bypassing gateway.

Figure 1 shows the most used device–gateway–cloud scheme in smart home. Usually, users need to tell the ssid–password pair to devices. The devices only need to check if they can connect to the particular Wi-Fi correctly and do not need to respond to users. Therefore, the configuration progress is single-direction. Since the devices are not on the Internet yet, devices and users cannot recognize each other. The result is that users can only broadcast the information that the devices need. Obviously, this single-direction broadcasting scheme is vulnerable to eavesdropping.

Some researchers have already evaluated several configuration strategies for Wi-Fi devices [16], whose target is to find what is the best method to transfer the Wi-Fi ssid and passphrase from a smartphone to a Wi-Fi device with little or no UI. They found that using USB or flashing (light sensor) suffers from platform dependency, and also indicated audio configuration is more platform-agnostic while requiring a cable and a few extra electronic components. However, most smart home devices are equipped with sonic devices.

### 2.2. Audio Wave Transmission in IoT

Audio waves have been used to transmit information for a long time, and acoustic sensor networks (ASNs) are gaining increasing attention [17]. Naratte indicated that its sound waves based transmission method is as fast as NFC but slower than Wi-Fi or Bluetooth. However, it almost has no “setup time”, i.e., there is no waiting around for a handshake to be established between devices. Using audio waves to transmit data has the following advantages: (1) it does not have strong platform dependency; (2) it is lightweight and suitable for short-time temporary communication; and (3) it is difficult for remote attackers to eavesdrop. Audio wave transmission is facilitating IoT recently. For example, Alipay has already launched a new payment system using sound waves to connect smartphones with vending machines [18]. Ref. [19] provided audio-based interaction technology that lets the user have full control over their home environment, detects distress situations and eases the social inclusion of the elderly and frail population. The “short distance feature” of audio transmission is also confirmed in previous research [20].

There are various techniques for transmitting data by audio waves. However, unlike Wi-Fi, audio wave transmission does not have international standards such as 802.11, thus manufacturing methods vary, which means it cannot be implemented simply and is much more difficult to be hacked than other techniques. In this paper, we propose a simple and secure scheme for transmitting data by audio waves, which mainly aims at the process configuring the network for IoT devices.

### 2.3. Present Configuration Methods

The Wi-Fi connection always relies on chips, thus chip manufacturers take the responsibility to provide a technology for configuration. TI is the first manufacturer to conquer this technology and its solution’s name is SmartConfig, after which other manufacturers have put forward their solutions successively. Researchers have surveyed the connection technology that manufacturers use [21]. We present them in Table 1.

All of these solutions are based on 802.11 packets, thus we take SmartCfg as the example to do the subsequent analysis and comparison.

#### SmartCfg

SmartCfg is a network distribution technology introduced by Texas Instruments (TI) in 2012 [22]. Its main function is to connect smart home devices to a Wi-Fi network without UI quickly. It is now widely adopted by many wireless chip manufacturers. It is based on broadcasting encoded authentication credentials of Wi-Fi networks within 802.11 packets. Because of the dynamic encryption of 802.11 packets, devices cannot decode the data field of a captured 802.11 packet. Thus, the credentials are always stored in meta data fields. SmartCfg has three methods to store credentials: (1) Data in Multicast Addresses (DMA), which means that credentials are hidden in the last 23 bits of the destination address field; (2) Data in Packet Length (DML), in which method encoded message is stored in length field; and (3) Hybrid, which takes use of both DMA and DPL.

Different chip manufacturers choose different methods and encoding schemes. When using SmartCfg, the device goes into SmartCfg state, sniffing all 802.11 packets that may be the credential of Wi-Fi. Users encode the ssid and the password as 802.11 packets using the related app. Progress finishes when the device successfully connects to the given Wi-Fi network.

According to the previous narrative, there are three main disadvantages in SmartCfg:

1. Although SmartCfg has three different storing schemes, it is still easy for attackers to detect which scheme the app is using because of the obvious features of these three schemes. The reason is that 802.11 packets have designed format. The positions of all meta data are public to all. As a result, SmartCfg is not secure enough congenitally.
2. Wi-Fi packet is easy to be eavesdropped as 802.11 packets have various frequency bands. To make sure the configuration progresses properly, the devices need to sniff all frequency bands in turn and the user’s app should send the same packets many times to ensure that no packet is lost, which increases the possibility that packets are captured by attackers.
3. According to the second disadvantage, it is hard for the devices to capture the correct packet quickly because they have to sniff all frequency bands one by one. That is, SmartCfg is not efficient enough.

### 2.4. Other Possible Techniques

#### 2.4.1. Bluetooth Low Energy

Bluetooth Low Energy (BLE) is another widely used wireless interconnection solution. BLE has a commendable speed to transmit credential of a wireless network after its pairing. The problem is that there are many security flaws in BLE, for example blueborne [23]. In addition, hackers can even steal data passing between devices using man-in-the-middle, attacking a decade-old BLE flaw [24]. Considering that bluetooth packets are also easy to be captured, using bluetooth to do the configuration is not a secure idea.

#### 2.4.2. Wi-Fi Direct

Wi-Fi Direct is a promising protocol that enables devices to easily connect with each other without requiring a wireless access point. It is efficient but Shen et al., [25] indicated Wi-Fi Direct is susceptible to security threats due to the open access of wireless channels and lack of security infrastructures, similar to SmartCfg.

## 3. Audio Wave Based Credential Transmission

### 3.1. Overview

In this section, we introduce our solution to credential transmission for devices without UI. This new solution uses audio waves based on MFSK16 [26] to conduct the transmission instead of 802.11 packets. We designed audio packet to take full use of the packets to prevent storing data in meta-data as in 802.11 methods. We do not use multi-frequency bands, but just use different frequencies to represent different characters. That is, devices only need to listen continuously and start decoding when a particular frequency is recognized. It also means this audio based solution has no efficiency problem as SmartCfg does. Furthermore, there are sounds everywhere in reality, and it is difficult for attackers to confirm which frequency the encoder is using. Besides, when attackers are a bit far away from the user and the device, it is very hard to eavesdrop what the app has sent a message.

### 3.2. Scheme Design

#### 3.2.1. Modulation

There are various ways to do the modulation. The most suitable method is to hide information within frequencies of audio waves, which is called FSK (Frequency-shift keying). It uses several frequencies representing different messages. The message to send will be modulated into a list of audio wave pulses, and each part has the same duration while belonging to one of the chosen frequencies.

**Figure 2:**
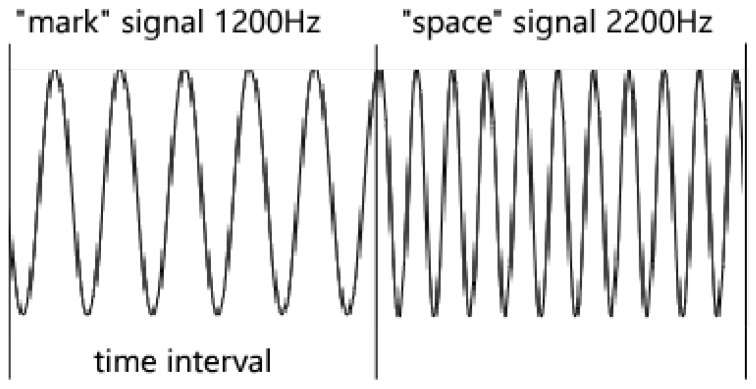
An example of modulations of audio waves used in Bell 202.

The first FSK method about using audio waves is AFSK, which has a standard called Bell 202 [27]. Bell 202 transfers data at the rate of 1200 bits per second using two frequencies representing for binary “0” and “1”. As Figure 2 shows, it uses a 2200 Hz tone to represent a “space” signal and a 1200 Hz tone to represent a “mark” signal, where mark is a binary bit 1 and space is 0.

There is also another modulation that uses more than two frequencies: MFSK. MSFK16 is a digital mode designed by amateurs for keyboard conversations at high frequencies. It is specifically designed to provide good performance over long paths and over polar DX paths. It is a semi-duplex non-arq continuous phase MFSK synchronous mode, with full forward error correction (FEC). Each tone emblems one multi-bit symbol, which generates digital representation of the 16 tones used in a phase-synchronous manner and then sends it to the sound components for conversion to audio.

We chose MFSK16 because it provides higher efficiency and greater accuracy. Transmitting by MFSK16 is straightforward. The data to send are stored in a buffer, and are sent via a series of coders to the transmit modulator once the transmitter is activated, which generates digital representation of the 16 tones used in a phase-synchronous manner. Then, the message is sent to the audio components for conversion.

Compared with other methods, MFSK16 has these advantages:

- It transmits fewer bits than other methods, which provides higher efficiency.
- It has high resistance to noise due to narrow receiver bandwidth per tone.
- It can hardly be affected by ionospheric effects such as Doppler, fading and multi-path.
- The error rate of MFSK family reduces as the number of tones is increased, and 16 tones is most suitable.

In MFSK16, each tone represents for 4 bits. Thus, $2^{4}=16$ tones can represent all possible values of 4 bits. According to our series of experiments, we chose 1200 Hz as the first tone representing for binary “0000”, and we chose 40 Hz as the interval frequency so that the 16 tones in our MFSK16 are 1200 Hz, 1240 Hz, … 1480 Hz. The represented bits set are arranged as gray code to reduce the error rate, because similar tones carrying similar bits can cause fewer errors than those carrying very different bits. Table 2 shows the representing pairs of tones and bits sets.

For example, binary value of character “A” is “01000001”, which will be divided into two parts “0100” and “0001”, where “0100” requires tone 1320 Hz and “0001” requires tone 1200 Hz. Therefore, if a character “A” is to be sent, two tones of 1320 Hz and 1200 Hz will be generated and stored into buffer.

#### 3.2.2. Packet Format

Under ordinary circumstances, to transfer message correctly, we always need to add a preamble for devices to recognize the particular messages. We also need some error correcting code (ECC) as insurance. The preamble’s intention is for smart devices to recognize that what they are receiving is the credential they are waiting for. However, we do not need to synchronize data in this Wi-Fi provisioning process, because the communication is short-term and sudden. In addition, the receiver is not listening unless the owner wants to configure its Wi-Fi connection. As a result, the preamble does not need to be long. We can just use one tone of 16 to inform the receiver that message has arrived.

**Figure 3:**
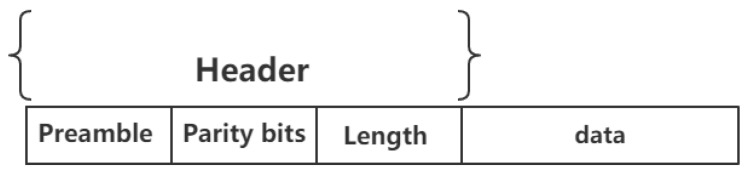
The format of audio wave packet.

As for error correcting code, we use advanced parity bits to make the scheme as lightweight as possible. Advanced parity bits means hamming weight of the message modulus 16, which can be represented by a tone. Besides, we believe the length of the message would not be too long for it contains only ssid and password for a wireless network. We are sure that the length is no longer than $2^{15}\times 4=$ 131,072 bits, thus we can use one tone to indicate how many tones are followed. Thus, the head of packet contains three tones, i.e., 12 bits. Figure 3 shows the format of audio wave packet.

### 3.3. Work Process

There are two parties in the configuration process, the sender (user with app) and the receiver (smart device). Now, we explain the process about how to accomplish the configuration.

#### 3.3.1. Signal Generation

The user inputs the ssid and password of a wireless network within the app, and then the generating process starts. There are two steps in this process. The generator does the encryption first, about which we give a detailed introduction below. Then, it generates a list of tones from the obtained cipher. After it gets the signal list, the app sends these signals (play sounds) several times, which is equivalent to the broadcasting that SmartCfg does.

#### 3.3.2. Signal Receiving

When listening mode starts, the smart device checks the sound all the time. The device is in wait-mode first. When it finds a particular tone (preamble) that belongs to the particular 16 tones, it changes into receiving mode. The receiver marks the incoming two tones as “head” and the followings as “data”. It uses non-coherent demodulation, using an FFT filter and demodulator technique, integrating the signal over the symbol tone period by sampling the period synchronously with the transmitted symbol. After that, it checks the parity bits. If there is no error, the device will try to connect to the Wi-Fi using decoded credential.

### 3.4. Encryption

Although we have a secure distance compared to other configuration methods, which means attackers are hard to eavesdrop the packets, we still need to do encryption for a higher security. Note that what we propose is an audio wave transmission framework, within which any reasonable encryption method could be used.

Our target is to find a secure encryption key that is difficult for others to attack. This must be based on a common secret between the user and the smart device. In a bad situation, attackers can get access to the device, and any information posted on the device is not secure. Suppose that, when the user buys a smart device, he gets a token from the manufacturer in some way and the token can only be certificated by the cloud server. In this situation, the user can use this token to get a common secret key with the device from cloud server, because the manufacturer can give every device a unique id when it is manufactured [2]. The unique id may not be long enough to be secure, which means we need further steps.

Based on the common secret, there are some methods we can take to do the key-exchange for later communication.

- Use a secure hash function *H*, and use the H(unique_id) as the key to do the encryption, such as SHA256. This method is the fastest and can deal with most situations. However, once the hash function used is found by attackers, it becomes very vulnerable.
- Use ECDH (Elliptic Curve Diffie-Hellman) to do the key-exchange [28]. This method is secure but it need the manufacturer to implement PKI (Public key infrastructure) on each of its devices, which increases the cost. In addition, the speed of ECDH is not fast enough.
- Use password-authenticated key exchange (PAKE) to establish a secure cryptographic key, such as J-PAKE (Password Authenticated Key Exchange by Juggling) [29], which is a fast PAKE protocol providing two or more parties to establish private and authenticated communication solely without PKI based on their shared (low-entropy) secret. In this method, the cryptographic key is computed before the configuration process. This method takes a little time to do the PAKE process but is very secure.

The first encryption method is effective and with a high security level, since sounds fade quickly when traveling over distance; adding a jamming signal is the best direct way to enhance the security of transmission, and the jamming signal rules are ready to be designed by users.

Each method introduced above is based on a shared secret, and we should consider the possibility that attackers can get the access to the device, which means they can get the UUID or QR-code posted on the device. To prevent this kind of attack, we assume that owner is always able to be close to the device but the attackers are not. Therefore, the ambient sound shared by owner and device can be used to strengthen security.

Previous work [30] has described a method for authentication based on ambient sound. Since we are transmitting data through sound waves, it is reasonable to obtain similar authentication methods. When configuring Wi-Fi, both user and device extract some information from ambient sound, by which the device can judge the reliability of received packet. Furthermore, information extracted from ambient sound can also be used to implement one-time pad, which is indestructible. However, it remains difficult for both device and user to generate exactly the same key stream in noisy environment. Therefore, ambient sound is only used for authentication thus far.

### 3.5. Summary

In this section, we propose a novel solution to Wi-Fi provisioning based on MFSK16, which transmits data using audio waves. We designed the packet format as lightweight as possible to improve user experience. We also introduced some encryption methods that can be implemented to provide higher security. In most cases, using a secure hash function is enough because Wi-Fi provisioning process does not happen frequently. Once the device is connected to Wi-Fi, Wi-Fi provisioning process will not happen for years.

## 4. Experimental Evaluation

### 4.1. Environment and Settings

**Figure 4:**
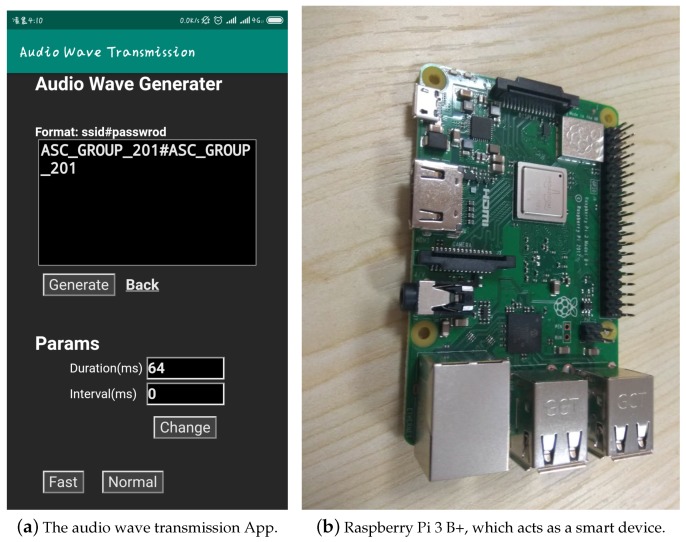
(**a**) The app can encode Wi-Fi credential into audio waves; and (**b**) the Raspberry Pi used to act as a smart device, which listen and decode audio waves.

We developed an android application to generate audio waves (Figure 4). We used a Raspberry Pi 3 B+ as the smart device to receive and decode audio waves. The experiments were carried out in a laboratory where there is always some noise.

The Wi-Fi ssid and password were random strings that have 7–20 bytes. We set an UUID (Universally Unique Identifier) to the smart device, and took H(uuid) as the keystream used for encryption, where *H* is SHA-256.

### 4.2. Results

Since our recognition is based on loudness of the sound and the loudness decreases with distance, our audio wave transmission has a short-distance effective feature, which is the reason for the secure-distance. We tested the audio wave based transmission solution at different distances to observe its success rate. We also counted the mean time that smart devices need to recognize the correct credential. The experiment result is shown below.

Precise rate is calculated using formula

$$precise\_rate=\frac{correct\_bits}{all\_bits}$$

As Table 3 shows, audio transmission works very well when the sender is close to the receiver. The success rate is 100%. When the mobile phone is a little far from the microphone, the success rate drops heavily in normal loudness. If the audio is relatively loud, the device can still usually get the right message. However, when the distance rises to 100 cm, the device can hardly decode any message with the normal loud audio and has a poor success rate with a louder sound.

Table 4 shows the mean time spent before the device recognized the credential correctly. We can see the time does not exceed 2 min, which is tolerable for users.

#### 4.2.1. Security Analysis

**Figure 5:**
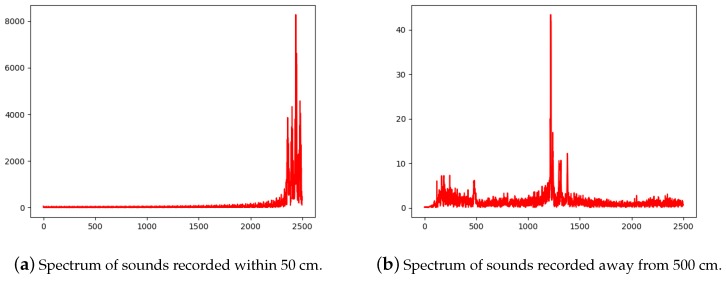
(**a**) The spectrum of sounds recorded very close to the device; and (**b**) the spectrum of sounds recorded a bit far from the device.

Considering that when we configured the wireless network for a smart device, we always set the credential correctly in the app and put the mobile phone near the device for it to receive. It means that the distance of smart device (receiver) and phone (sender) is always very close. We can ensure that the configuration process will execute smoothly. Moreover, the experiment result also points that it is almost impossible for attackers to eavesdrop the audio wave message. We also used recorder to record the sound when carrying out experiments. The result is shown in Figure 5.

Figure 5b indicates that, when an attacker tries to eavesdrop the audio wave packets, he will get a terrible spectrum that can be hardly analyzed. Suppose an attacker cannot do suspicious action under the owner’s eye. Additionally, we can equip jamming signal to disturb the eavesdrop. Wi-Fi, ZigBee and Bluetooth all can go through walls and have a much farther transmission distance, whereas going through walls needs very loud sound, which would not appear in our audio waves transmission.

#### 4.2.2. Speed Analysis

We set the duration of each tone as 64 ms, thus this audio based method transfers data at the rate of about $\frac{4}{0.064}=62.5$ bits per second. Assuming that a whole packet including Wi-Fi ssid and password and the header is 30 bytes, it needs $\frac{30\times 8}{62.5}=3.84$ s to finish the network configuration. Although the success rate is 100% in our experiments, we still consider there is possibility that it needs several times to finish the configuration correctly. We still can finish the configuration in about 10 s (Table 4).

**Figure 6:**
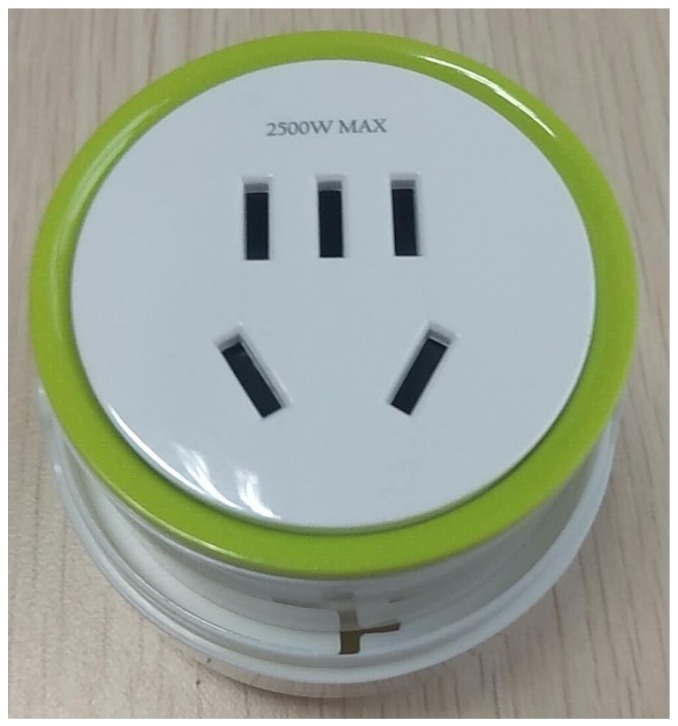
Konke’s smart socket.

To compare with 802.11 packets based Wi-Fi configuration methods, we carried out experiments on a Konke’s smart socket Figure 6. As shown in Table 5, it costs about 8 s to complete the configuration process, which is three times slower than our audio wave based method. Another conclusion of this experiment is that transmission accuracy using 802.11 packets is hardly affected by distance.

### 4.3. Summary

Compared to 802.11 methods, our audio wave based transmission has a higher speed and can effectively prevent eavesdropping because of its “short distance” feature. However, the “short distance” feature may also bring some problems; for example, a noisy environment could lead to DoS (Denial of Service) attack. However, considering Wi-Fi provisioning process only happens once when a user buys a smart device, these kinds of disadvantages can be easily avoided.

## 5. Discussions

The short distance message transmission based on audio waves still requires much work. For example, the baud rate can be greatly improved by using more accurate recognizable algorithms and better packet design. It can be widely used in short distance communication, and it would be more suitable if encryption were not necessary. Secure pairing for wearables is a problem of IoT, and present methods seldom obtain high efficiency [31]. Thus, using audio wave transmission on secure pairing for wearables is promising.

There are still many challenges for acoustic technologies. For example, transmission based on audio may not work well in a very noisy environments. There are some attacks aimed at audio waves, which are used as commands for smart devices [32]. Extracting information from acoustic side channels also has more and more effective experiments recently: Genkin et al., [33] proposed a new acoustic cryptanalysis key extraction attack applicable to GnuPG’s implementation of RSA. Genkin et al., [34] proved acoustic noises emanating from within computer screens can be used to detect the content displayed on the screens. Establishing a more complete audio transmission system that can transmit message at a considerable speed while keeping the data correct and resisting various attack is the next step.

## 6. Conclusions

Our work consists of an audio wave based anti-eavesdrop and fast short distance message transmission method and its application on Wi-Fi configuration for smart devices, the latter being introduced in detail. Through our experiments, we obtained the result that this new audio based method has higher security by having a very short effective distance. It also has a good transmit speed, for which there is a lot of room to improve. Compared to traditional 802.11 based configuration solutions such as smartCfg and some other possible solutions such as bluetooth, our method is more secure when in the configuration.

## Figures and Tables

**Table 1:** Solutions of manufacturers.

Manufacturer	Technique
TI	SmartConfig
MTK	SmartConnection
Marvell	EasyConnect
Reltek	SimpleConfig
Espressif	SmartConfig
Wechat	AirKiss

**Table 2:** 16 different tones in proposed MFSK16.

Tone (Hz)	Bits
1200	0000
1240	0001
1280	0011
1320	0010
1360	0110
1400	0111
1440	0101
1480	0100
1520	1100
1560	1101
1600	1111
1640	1110
1680	1010
1720	1011
1760	1001
1800	1000

**Table 3:** Precise rate at different distances.

	Normal Loudness	Relatively Loud
10 cm	100%	100%
30 cm	93.3%	100%
50 cm	72.5%	96.2%
100 cm	9.45%	42.6%

**Table 4:** Mean time spent (seconds) by audio wave transmission framework before the device recognized the credential correctly.

	Normal Loudness	Relatively Loud
10 cm	2.6	2.8
30 cm	2.8	2.6
50 cm	9.3	4.5
100 cm	95.2	64.2

**Table 5:** Mean time spent (seconds) by Konke’s smart socket before the device recognized the credential correctly.

Distance	Time Spent
10 cm	6.58
30 cm	6.19
50 cm	8.12
100 cm	8.57
